# Meta-Analysis of Catheter Ablation versus Medical Therapy for Heart Failure Complicated with Atrial Fibrillation

**DOI:** 10.1155/2021/7245390

**Published:** 2021-12-06

**Authors:** Xi Zhu, Yingbiao Wu, Zhongping Ning

**Affiliations:** Department of Cardiology, Shanghai University of Medicine & Health Sciences Affiliated Zhoupu Hospital, Shanghai 201318, China

## Abstract

**Objective:**

To compare the efficacy of catheter ablation and medical therapy in patients with heart failure and atrial fibrillation.

**Methods:**

We searched randomized controlled trials comparing catheter ablation versus medical therapy for heart failure and atrial fibrillation through PubMed, MEDLINE, Embase, Cochrane Clinical Trials Database, Web of Science, and China National Knowledge Infrastructure. Articles were investigated for their methodological quality using the Cochrane Collaboration risk of the bias assessment tool. Forest plots, funnel plots, and sensitivity analysis were also performed on the included articles. Results were expressed as risk ratio (RR) and mean difference (MD) with 95% confidence intervals.

**Results:**

Nine (9) studies were included in this study with 1131 patients. Meta-analysis showed a reduction in all-cause mortality from catheter ablation compared with medical therapy (RR = 0.53, 95% CI = 0.37 to 0.76; *P*=0.0007) and improved left ventricular ejection fraction (LVEF) (MD = 6.45, 95% CI = 3.49 to 9.41; *P* < 0.0001), 6-minute walking time (6MWT) (MD = 28.32, 95% CI = 17.77 to 38.87; *P* < 0.0001), and Minnesota Living with Heart Failure Questionnaire (MLHFQ) score (MD = 8.19, 95% CI = 0.30 to 16.08; *P*=0.04).

**Conclusion:**

Catheter ablation had a better improvement than medical treatment in left ventricular ejection fraction, cardiac function, and exercise ability for atrial fibrillation and heart failure patients.

## 1. Introduction

Atrial fibrillation (AF) and heart failure (HF) are common cardiovascular diseases in the 21st century [1]. The incidence rate of AF is positively correlated with age, especially for elderly people; the prevalence rate in people over 80 years of age is 9%∼15% [2]. AF can aggravate the risk of deterioration of heart function in patients with HF, accelerate the occurrence time of HF symptoms, and lead to severe limitation of daily activities and decline in quality of life [3, 4].

AF usually coexists with HF. There is a close pathophysiological relationship between them, including cardiac fibrosis and neurohumoral activation [5]. When AF occurs, hemodynamic changes, loss of effective atrial contraction, and rapid but irregular ventricular rate lead to reduced cardiac output and left ventricular dysfunction. In patients with HF, increased left ventricular filling pressure and atrial dilatation lead to structural and electrical remodeling, which may increase the risk of AF. The mortality of patients with simple HF or AF was significantly lower than that of patients with both AF and HF [6, 7].

Therefore, it is necessary to treat patients with HF and AF. However, some studies have shown that compared with ventricular rate control, the rhythm control of antiarrhythmic drugs in patients with AF combined with HF cannot effectively reduce the mortality of this population [8, 9]. The previous report shows that it is difficult to maintain the sinus rhythm with drugs and direct current cardioversion. In addition, antiarrhythmic drugs also have arrhythmogenic effects. Their arrhythmogenic effects will aggravate the HF of patients, so the benefits of drugs by converting to sinus rhythm will also be offset by this arrhythmogenic effect [10, 11]. Therefore, for this kind of patient, the choice of antiarrhythmic drugs for maintaining sinus rhythm has great limitations [12, 13].

With the maturity of radiofrequency ablation technology, the treatment strategy of converting and maintaining the sinus rhythm by radiofrequency ablation has become another choice for patients with AF to improve cardiac function [14]. It has been widely used clinically and proven to be safe and effective for patients with AF. With the further study of the mechanism of AF, new ablation technologies (including new ablation energy: cryotherapy, high-frequency ultrasound, laser, etc.) are emerging [15, 16]. Continuous improvement of ablation methods and devices, combined with drug therapy and minimally invasive surgery or with other comprehensive measures, will increase the benefits for patients with AF [17]. In this study, we conducted a meta-analysis on the randomized controlled trials of catheter ablation (CA) and traditional medical therapy in treating patients with HF and AF to provide a reference for clinical practice.

## 2. Methods

### 2.1. Literature Search Strategy

We will systematically search the relevant randomized controlled trials in the 6 databases from inception to June 2021, including PubMed, MEDLINE, Embase, Cochrane Clinical Trials Database, Web of Science, and China National Knowledge Infrastructure. We used the following keywords: (1) atrial fibrillation; (2) heart failure; (3) catheter ablation; and (4) medical therapy. Several times, the search strategy was refined by combining different keywords using the Boolean operators “AND” and “OR.” Our literature search was comprehensive, with neither language restrictions nor publication status limitations. To maximize the specificity and sensitivity of the search, the author should also check the reference list of the searched research to seek other relevant research that was not found through the search strategy.

### 2.2. Study Selection

The relevant articles were reviewed fully, ensuring the following criteria are satisfied: (1) inclusion only of patients diagnosed with AF and HF; (2) comparison of CA and medical therapy; and (3) complete experimental and control data.

The study was excluded based on the following predetermined exclusion criteria: (1) research not meeting the inclusion criteria; (2) the outcomes of interest were not reported or impossible to use; and (3) review, abstract, and duplicate publication.

### 2.3. Data Extraction and Quality Assessment

Titles and abstracts of all publications identified through the search were independently screened for inclusion by two authors. The following variables were summarized in a preformatted spreadsheet: authors, year of publication, characteristics of study participants (age and sex), study design, treatment approach, and primary outcome. A risk-of-bias assessment was conducted using the Cochrane Collaboration tool. In addition, the outcome indicators included all-cause mortality, left ventricular ejection fraction (LVEF), 6-minute walking time (6MWT), and the score of Minnesota Living with Heart Failure Questionnaire (MLHFQ). After screening the indicators, we found that the value of each indicator had much difference.

### 2.4. Statistical Analysis

Two authors independently used Review Manager (version 5.4, Nordic Cochrane Centre) to analyze all the data. To measure the consistency of the effect size (RR and MD), pairwise meta-analyses were performed with a DerSimonian and Laird random-effects model to calculate the pooled estimates of RR and MD with 95% CIs of direct comparisons between the CA group and medical therapy group. Continuous variables were expressed by MD and discontinuous variables by RR. Heterogeneity between and within designs was assessed using Cochran's *Q* and quantified using *I*^2^ statistics. *I*^2^ values less than 25%, 25% to 75%, and greater than 75% represented low, moderate, and high degrees of heterogeneity, respectively. Based on the absence or presence of significant heterogeneity, a fixed- or random-effects model was used. Sensitivity analysis was further conducted to evaluate the robustness of the findings through exponential tilting. Potential publication bias was assessed by visual examination of a funnel plot along with Egger's test for small-study effects.

## 3. Results

### 3.1. Search Process

**Figure 1:**
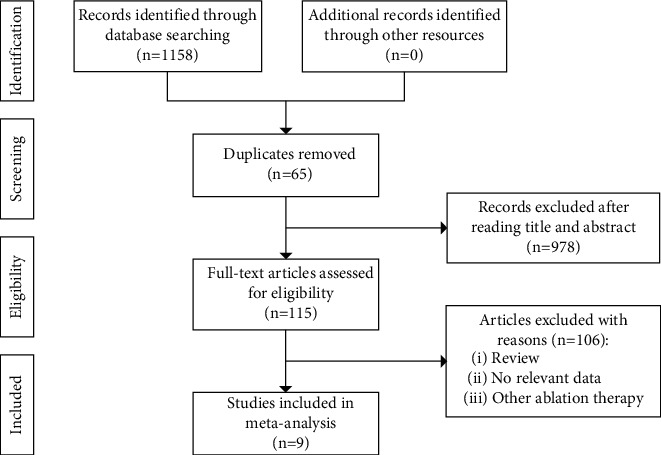
Flowchart showing the study selection process.

A total of 1093 potentially eligible studies were identified. After excluding 978 manuscripts that did not meet the inclusion criteria by reading the title and abstract, 115 full-text articles remained. One hundred and six (106) articles were excluded from further screening due to not satisfying the research direction and insufficient data and article type. Thus, nine (9) studies met the inclusion criteria and were included in the present meta-analysis [18–26].  Figure 1 shows the details of the systematic process for our literature search and selection process.

### 3.2. Characteristics of Included Studies

A total of 1131 patients were included in this meta-analysis. All articles were published from 2011 to 2019. These studies contained 9 RCTs involving 1131 patients, of which 564 received CA and 567 received medical therapy. The primary outcome contained left ventricular ejection fraction (LVEF), 6-minute walk test (6MWT), the score of Minnesota Living with Heart Failure Questionnaire (MLHFQ), all-cause mortality, and complications. The main inclusion study characteristics are summarized in Table 1.

### 3.3. Results of Quality Assessment

**Figure 2:**
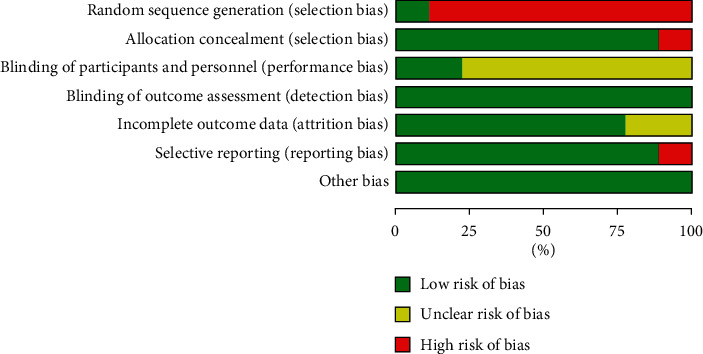
Quality assessment of the included studies: low (green hexagons), unclear (yellow hexagons), and high (red hexagons).

**Figure 3:**
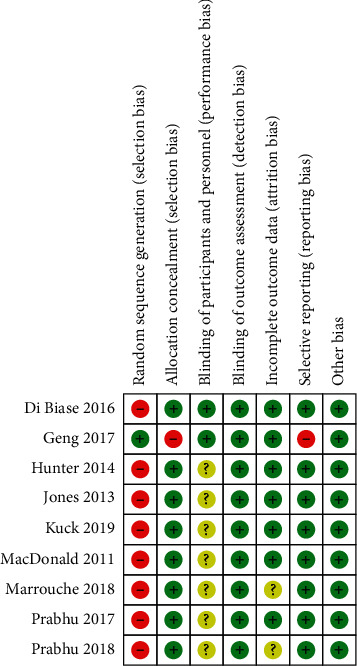
Summary of risk of bias of included studies.

Study quality was assessed using the Cochrane risk-of-bias tool. Among the 9 articles, as the risk of atrial fibrillation and heart failure, studies were often unable to be completely blind and randomized [27], so high risk of selection bias existed in most included articles; in addition, high risk of reporting bias and selection bias of allocation were found in one study (Figure 2). A summary of the risk-of-bias assessment for each study is shown in Figure 3.

### 3.4. Results of the Heterogeneity Test

**Figure 4:**
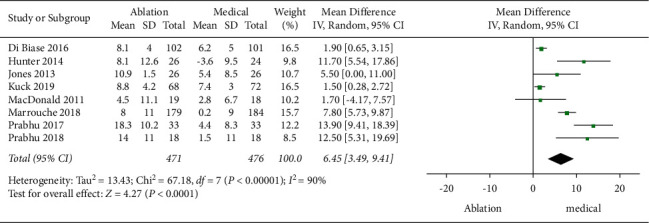
Forest plot of CA versus medical therapy: LVEF.

For LVEF, 8 studies involving 947 patients reported it. Meta-analysis showed that compared to the medical therapy group, the CA group had a higher increase of LVEF (MD: 6.45, 95% CI [3.49, 9.41], *P* < 0.0001, random-effects model), with significant heterogeneity (*P* < 0.0001, *I*^2^ = 90%) (Figure 4). We performed a sensitivity analysis by removing any included study, and the result did not change, suggesting it was robust.

**Figure 5:**
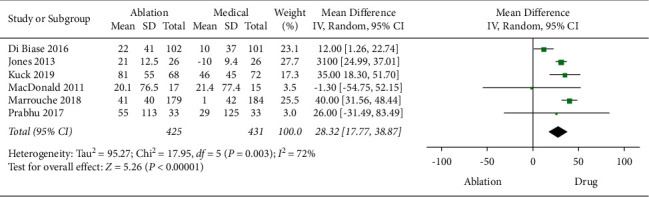
Forest plot of CA versus medical therapy: 6MWT.

In terms of 6MWT, 6 studies involving 856 patients contributed to the analysis. A random-effects model was used to evaluate the heterogeneity of 6MWT due to the significant heterogeneity (*P*=0.003, *I*^2^ = 72%). The pooled analysis showed that the CA group had a better improvement than the medical therapy group (MD: 28.32, 95%CI [17.77, 38.87], *P* < 0.0001) (Figure 5). The result was not changed significantly after sensitivity analysis.

**Figure 6:**
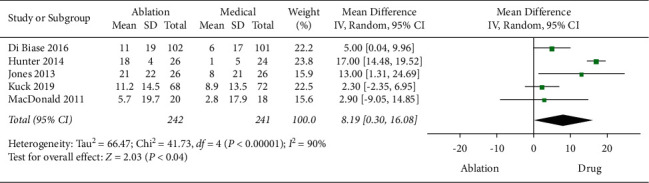
Forest plot of CA versus medical therapy: MLHFQ score.

On the increase of MLHFQ score, 5 studies were included for analysis. An overall mean difference of 8.19 between the CA group and medical therapy group (95% CI = 0.30 to 16.08), with statistical significance (*P*=0.04), was found (Figure 6). The comparisons presented a high heterogeneity among included studies (*P* < 0.0001 and *I*^2^ = 90%); however, sensitivity analysis showed the result was stable.

**Figure 7:**
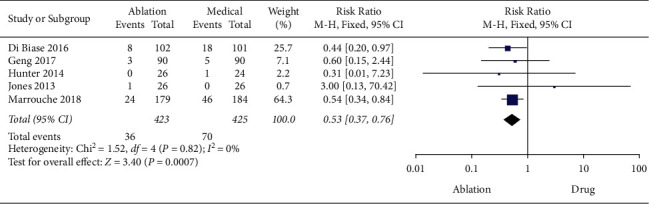
Forest plot of CA versus medical therapy: all-cause mortality.

Five studies reported all-cause mortality. A fixed-effects model was used to evaluate the heterogeneity of all-cause mortality owing to the homogeneity among included studies (*P*=0.82, *I*^2^ = 0%). The results showed that the all-cause mortality in the CA group was significantly lower than in the medical therapy group (RR = 0.53 with 95%CI 0.37 to 0.76, *P*=0.007) (Figure 7).

**Figure 8:**
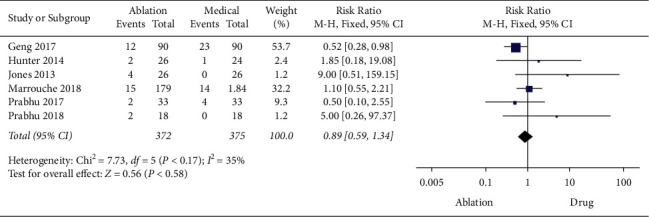
Forest plot of CA versus medical therapy: complications.

Similarly, a fixed-effects model was adopted to evaluate the heterogeneity of complications as the moderate heterogeneity among included studies (*P*=0.17, *I*^2^ = 35%). The results showed no significant difference between the CA group and the medical therapy group in a pooled analysis of complications (RR = 0.89 with 95% CI 0.59 to 1.34, *P*=0.58) (Figure 8).

### 3.5. Publication Bias

**Figure 9:**
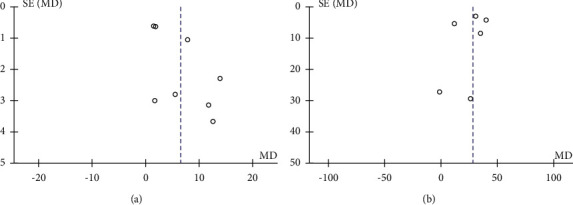
Funnel plot for potential publication bias.

Potential publication bias was assessed by a funnel plot and Egger's linear regression test. The shape of the funnel plots showed some evidence of symmetry (Figure 9), and Egger's test was not significant (LVEF *P*=0.535; 6MWT *P*=0.487), which indicated no significant publication bias existed in these results.

## 4. Discussion

Catheter ablation (CA) is a kind of interventional therapy for tachyarrhythmia. It has been used in the clinic for more than 30 years since 1987 [28]. It is an interventional technique that the electrode catheter is delivered to a specific part of the cardiac cavity via the vein or artery to release radiofrequency current, leading to coagulation necrosis of local endocardium and subendocardial myocardium, to inhibit the abnormal conduction bundle and origin point of tachyarrhythmia [29]. This technique has the advantages of no operation, small trauma, and a high success rate. It has brought revolutionary changes to the treatment of tachyarrhythmia. It has become the first choice for radical treatment of atrioventricular reentrant tachycardia, atrioventricular nodal reentrant tachycardia, atrial tachycardia, atrial flutter, and idiopathic, and bundle branch reentrant tachycardia [30, 31]. For patients with simple AF cardiomyopathy, early CA can achieve the cure level. Patients with impure AF cardiomyopathy can also improve heart function, improve quality of life, and reduce hospitalization rate [32].

Our study aimed to evaluate the effect of CA on cardiac function in patients with AF and HF. After summarizing and analyzing 9 randomized controlled trials involving 1131 patients, we found that CA significantly improved patients' left ventricular ejection fraction (LVEF) (MD = 6.45, 95% CI = 3.49 to 9.41; *P* < 0.0001), 6-minute walking time (6MWT) (MD = 28.32, 95% CI = 17.77 to 38.87; *P* < 0.0001), and MLHFQ score (MD = 8.19, 95% CI = 0.30 to 16.08; *P*=0.04) and reduced all-cause mortality (RR = 0.53, 95% CI = 0.37 to 0.76; *P*=0.0007) compared with conventional medical therapy (rhythm control, heart rate control, or a combination of both). The sample size was large, and the results were relatively stable, providing a certain basis for clinical decision making. Our results had high heterogeneity, which may be attributed to the limited included articles. We could conduct further analysis with more eligible researchers to avoid high heterogeneity in the future.

Long-term follow-up may lead to many lost follow-ups when evaluating the effects of different treatments on cardiac function [33]. Secondly, patients' heart function may be disturbed by other factors such as other diseases, age, and living habits [34]. Therefore, in our analysis, we limited the results of outcome variables to the maximum follow-up time of two years to reduce the impact of the abovementioned factors on cardiac function and more accurately reflect the treatment effect.

Compared with antiarrhythmic drugs, CA can more effectively maintain the sinus rhythm in patients with AF, making it the first-line treatment recommended by the guidelines [2]. However, the success rate of ablation in patients with HF is still low. Most patients needed at least two ablation operations to maintain the sinus rhythm [4] effectively. The change of heart structure made it more difficult to achieve complete pulmonary vein isolation. For these reasons, CA has not been recommended as class I in current guidelines [5]. Recent studies and meta-analyses have compared the related complications between ablation and medical therapy, and the results show no significant difference, which determined the safety of CA [33, 35]. This meta-analysis summarized all the current randomized controlled trials, confirmed the advantages of CA in improving cardiac function, and provided a more favourable basis for clinical decision making.

As far as radiofrequency CA is concerned, it is suggested that patients with symptomatic and refractory AF should receive radiofrequency CA [36, 37]. In our study, we focused on patients with AF and HF. CA is considered the treatment of HF to a large extent rather than AF. Compared with drug therapy, CA has many factors, such as operation risk, operation failure, and high cost [32].

To obtain the best risk-benefit ratio, it is necessary to select suitable patients for CA. Studies have shown that the success rate and benefit degree of CA in patients with AF and HF are related to the size of the left atrium, the load and duration of AF, the primary heart disease of HF, and the degree of the myocardial lesion, among which the left atrial matrix is closely related to the success of CA in patients with AF [38, 39]. For patients with AF cardiomyopathy, especially for patients with AF dilation, the treatment strategy should go hand in hand with the treatment of HF and AF [40]. Because the disease is secondary to AF cardiomyopathy, removing the aetiology can effectively treat the disease, and early CA can reach the cure level. Patients with impure AF cardiomyopathy can also significantly improve heart function, improve quality of life, and reduce hospitalization rate [41].

There were many limitations in this study. First, the measurement of LVEF in this study was not unified, especially the use of echocardiography, which influenced technical and subjective factors, and the results were not objective. Second, the follow-up time of the included studies was different; 4 were 6 months, the rest were 12 months and 24 months, respectively. Third, although all the included studies met the inclusion criteria, the degree of HF and duration of AF were not strictly limited.

In this study, we conducted a meta-analysis of 9 randomized controlled trials involving 1131 patients. Finally, we concluded that compared with medical therapy for atrial fibrillation and heart failure patients, catheter ablation therapy could improve left ventricular ejection fraction, cardiac function, and exercise ability and reduce mortality.

## Figures and Tables

**Table 1:** Characteristics of patients included in this study.

Study	Country	Study design	Treatment approach		No. of patients		Gender (M/F)		Age		Follow-up (months)	Primary outcome
			Ablation	Medical	Ablation	Medical	Ablation	Medical	Ablation	Medical		
MacDonald, 2011	UK	RCT	PVI and substrate modification	*β*-blockers and/or digoxin	22	19	17/5	15/4	62.3 ± 6.7	64.4 ± 8.3	>6	LVEF, 6MWT, MLHFQ
Jones, 2013	UK	RCT	PVI and substrate modification	*β*-blockers and/or digoxin	26	26	21/5	24/2	64 ± 10	62 ± 9	12	LVEF, 6MWT, MLHFQ, mortality, complications
Hunter, 2014	UK	RCT	PVI and substrate modification	*β*-blockers	26	24	25/1	23/1	55 ± 12	60 ± 10	12	LVEF, MLHFQ, mortality, complications
Di Biase, 2016	US	RCT	PVI and substrate modification	Amiodarone	102	101	77/25	74/27	47 ± 4.2	48 ± 4.9	24	LVEF, 6MWT, MLHFQ, mortality
Geng, 2017	China	RCT	PVI and substrate modification	*β*-blockers and/or digoxin	90	90	45/45	41/49	64.7 ± 9.4	65.4 ± 11.4	12	Mortality, complications
Prabhu, 2017	Australia	RCT	PVI and substrate modification	*β*-blockers and/or digoxin	33	33	31/2	29/4	59 ± 11	62 ± 9.4	6	LVEF, 6MWT, complications
Marrouche, 2018	US	RCT	PVI and substrate modification	Rate or rhythm control	179	184	156/23	155/29	64 (56 − 71)	64(56 − 73.5)	>6	LVEF, 6MWT, mortality, complications
Prabhu, 2018	Australia	RCT	PVI and substrate modification	*β*-blockers and/or digoxin	18	18	—	—	59 ± 13	63 ± 7.1	6	LVEF, complications
Kuck, 2019	Germany	RCT	PVI and substrate modification	Rate or rhythm control	68	72	60/8	66/8	68 ± 8	65 ± 8	12	LVEF, 6MWT, MLHFQ

RCT = randomized controlled trial; PVI = pulmonary vein isolation; LVEF = left ventricular ejection fraction; 6MWT = 6-minute walk test; MLHFQ = Minnesota Living with Heart Failure Questionnaire.

## Data Availability

The data are available in text uploaded with the manuscript.
